# Accidental injuries in the Pediatric Emergency Department

**DOI:** 10.1186/1865-1380-7-S1-P9

**Published:** 2014-07-25

**Authors:** Sarath Parambil Sasidhara Kurup, Swathi Padankatti, Kuruvilla Thomas

**Affiliations:** 1 Sundaram Medical Foundation Dr. Rangarajan Memorial hospital, Shanthi Colony, Anna Nagar, Chennai, India

## Objective

To study the spectrum of accidental injuries in the Pediatric Emergency Department of a community hospital.

## Methods

Setting: Pediatric Emergency Department (ED) of a Community hospital with around 10500 ED visits annually. The hospital conducts DNB Pediatrics, MCEM and BSc Accident & Emergency Technology Courses.

Participants: 784 Children with accidental injuries between 0 and 18 years of age who attended ED in a 10-month period from January 2013 to October 2013. Design: Retrospective observational study.

Tools: The ED records of the study group were analysed retrospectively.

## Results

Of a total of 8855 children seen in the ED during the study period, 784 (8.9%) presented with accidental injuries. The age distribution was 48 (6.1%), 345 (44%), 172 (21.93%), 131 (16.71%), 88 (11.2%), in <1 year, 1-5 years, 5-10 years, 10-15 years and 15-18 years age groups respectively. Falls accounted for 492 (62.8%), cut injuries 76 (9.7%), RTA 60 (7.7%), poisoning 48 (6.1%), sports related injuries 39 (4.97%), burns 32 (4.1%), crush injuries 15 (1.91%), foreign body 12 (1.53%), fight with peers 8 (1.02%), child abuse 2 (0.25%).

In 495 (63.1%) males, falls accounted for 307 (62.4% of total), cut injury 56 (73.7%), RTA 37 (61.7%), poisoning 25 (52.1%), sports related injuries 28 (71.8%) burns 18 (56.3%), crush injuries 7 (46.7%), foreign body 7 (58.3%), fight with peers 8 (100%), child abuse 2 (100%).

Majority of accidents occurred in evening hours (290, 36.9%) and at home (457, 58.2%). Of 552 falls and RTAs, 115 (20.8%) and 86 (15.6%) sustained head injury and extremity fracture respectively; 3 (2.6%) and 18 (21%) required immediate surgical intervention and open reduction respectively.

232 (29.6%) cases required hospitalisation. There was one death (a 15-year old girl who fell from terrace). Falls were the major cause of injury irrespective of age and gender.

## Limitations

A longer study period could have perhaps yielded a larger spectrum of injuries.

## Conclusion

Childhood accidental injuries can cause serious morbidity and mortality. Injuries peaked in the 1-5 years age group with falls being the leading cause of injury. Studying epidemiology of childhood injuries can help formulate effective preventive strategies and increase parental awareness.

